# Cyclooxygenases in pancreatic ductal adenocarcinoma: are they inflammatory catalysts for tumor growth

**DOI:** 10.3389/fonc.2026.1916208

**Published:** 2026-08-06

**Authors:** Adile Orhan, Tugba Orhan, Hans Raskov, Ismail Gögenur

**Affiliations:** 1 Center for Surgical Science, Department of Surgery, Zealand University Hospital, Koege, Denmark; 2 Faculty of Health and Medical Sciences, University of Copenhagen, Copenhagen, Denmark

**Keywords:** COX inhibitors, COX-2, cyclooxygenase enzymes, inflammation, metastasis, PDAC, PGE2

## Abstract

Pancreatic ductal adenocarcinoma (PDAC) remains one of the most lethal cancers with a relative 5-year survival around 12%. One of the greatest challenges in PDAC is the frequent systemic dissemination at the time of diagnosis and the dense, fibrotic extracellular matrix that prevents chemotherapeutics and cytotoxic immune cells from entering the tumor stroma. The cyclooxygenase (COX) enzymes and their downstream molecular products play important roles in cancer development and dissemination. The COX isoenzymes are overexpressed in many cancers and inhibition of the COX-2 isoform, and the use of acetylsalicylic acid has recently been demonstrated to reduce cancer recurrence after surgery in subgroups of patients with certain molecular subtypes, such as PI3K mutations, overexpression of vascular growth factors, etc.). COX-2-related pathways are involved in suppressing tumor immunity and promoting stem cell-like properties of cancer cells. Especially in PDAC, targeting the COX enzymes might be of importance in subgroups or as an adjunct as COX-2 is frequently overexpressed and associated with a worse prognosis. Selective COX-2 inhibitors have been demonstrated to reduce tumor growth, invasiveness, and metastasis formation in preclinical trials. This review evaluates existing preclinical and clinical studies investigating the potential for targeting the COX enzymes in PDAC treatment.

## Highlights

- COX-2 is overexpressed in up to 90% of all PDAC cases, while virtually absent in healthy pancreatic tissue. This makes the enzyme a central marker for disease progression as the enzyme contributes to the epithelial-to-mesenchymal transition and metastasis formation.
- The importance of COX-1 in cancer is largely unknown and research in the area is warranted.
- Prostaglandin E_2_ is a catalyst for tumor growth by promoting cell proliferation, inhibiting apoptosis, and stimulating angiogenesis.
- COX-2 and PGE2 contributes to an immunologically “cold” tumor microenvironment by suppressing the activity of cytotoxic T-cells and NK cells. Regulatory T-cells and myeloid-derived suppressor cells are recruited to shield the tumor from the immune system.
- This review highlights that while monotherapy with COX inhibitors (such as NSAIDs or Celecoxib) is rarely sufficient, clinical data suggest a significant potential in combination therapies - particularly alongside chemotherapy (e.g., Gemcitabine) or as a preventive measure post-surgery to reduce recurrence.

## Introduction

Pancreatic cancer encompasses both (1) exocrine cancers (adenocarcinoma, acinar cell carcinoma, adenosquamous carcinoma, and colloid carcinoma) and neuroendocrine cancers (insulinomas, gastrinomas, and nonfunctioning). Among all pancreatic cancers, the exocrine pancreatic ductal adenocarcinoma (PDAC) is by far the most prevalent, representing more than 90% of all cases while remaining the fourth leading cause of cancer death in both men and women in the USA (1). Globally, PDAC is responsible for around 8% of all cancer-related deaths (1). PDAC remains one of the most challenging and lethal cancers, primarily because of advanced stage at the time of diagnosis. The invasive nature of the cancer and the rapid formation of micro-metastases are some of the greatest challenges (2).

The incidence of PDAC has increased since 1990 and it is estimated that it will further rise in the following years (1, 3), emphasizing the urgent need for research to increase the cure rate. One of the greatest challenges regarding PDAC is early dissemination. At the time of diagnosis, around 75% of the patients present with stage III-IV disease, resulting in only 20-25% of patients being suitable for surgery (3). Genes promoting early migration and dissemination are the focus of intense research.

Irrespective of the nature of carcinogenesis, inflammatory cells and inflammatory mediators.

are detected in most tumor tissue. It is well known that inflammation acts on tumor cells and stromal cells and contributes to a tumor-promoting microenvironment. Persistent inflammatory stimuli promote genomic instability, oxidative DNA damage, epigenetic alterations, and dysregulated tissue repair, creating a microenvironment that supports malignant transformation (4–6). In addition, chronic inflammation stimulates the production of cytokines, chemokines, growth factors, and lipid mediators that promote cell proliferation, angiogenesis, immune evasion, and extracellular matrix remodeling (4–6). Within this inflammatory milieu, cyclooxygenase-2 (COX-2) is frequently upregulated and contributes to tumor initiation and progression through increased prostaglandin synthesis, particularly prostaglandin E_2_ (PGE_2_), which modulates both tumor cells and the surrounding stromal and immune compartments. These observations support the concept that chronic inflammation is not merely a consequence of tumorigenesis but an active driver of PDAC development and progression.

Research has demonstrated that COX-2 is upregulated in many human cancers including breast (7), prostate (8), lung (9), and PDAC (10) and there is a rich body of evidence suggesting that both isoenzymes, COX-1 and COX-2, are involved in multiple aspects of cancer pathophysiology. In most cases, these enzymes seem to operate in a coordinated manner.

Here, we review preclinical and clinical studies on COX expression in PDAC and discuss future directions for multimodal treatment opportunities.

## Evidence acquisition

This study was conducted as a narrative review. Literature searches were performed in PubMed using predefined search terms. Search terms included combinations of “pancreatic cancer,” “pancreatic ductal adenocarcinoma,” and “cyclooxygenase”. Additional studies were identified through manual screening of reference lists from relevant publications. Controlled vocabulary (MeSH) terms were not systematically applied, as the objective was to identify representative literature rather than perform a comprehensive systematic search.

Studies were selected based on their relevance to the scope of the review, with emphasis placed on original research articles, clinical studies, and major review papers addressing the role of cyclooxygenase genes in pancreatic cancer. The findings were synthesized descriptively to provide an overview of current knowledge and emerging perspectives in the field. The literature search was last updated in April 2026.

## Cell membrane phospholipases and arachidonic acid metabolism

**Figure 1:**
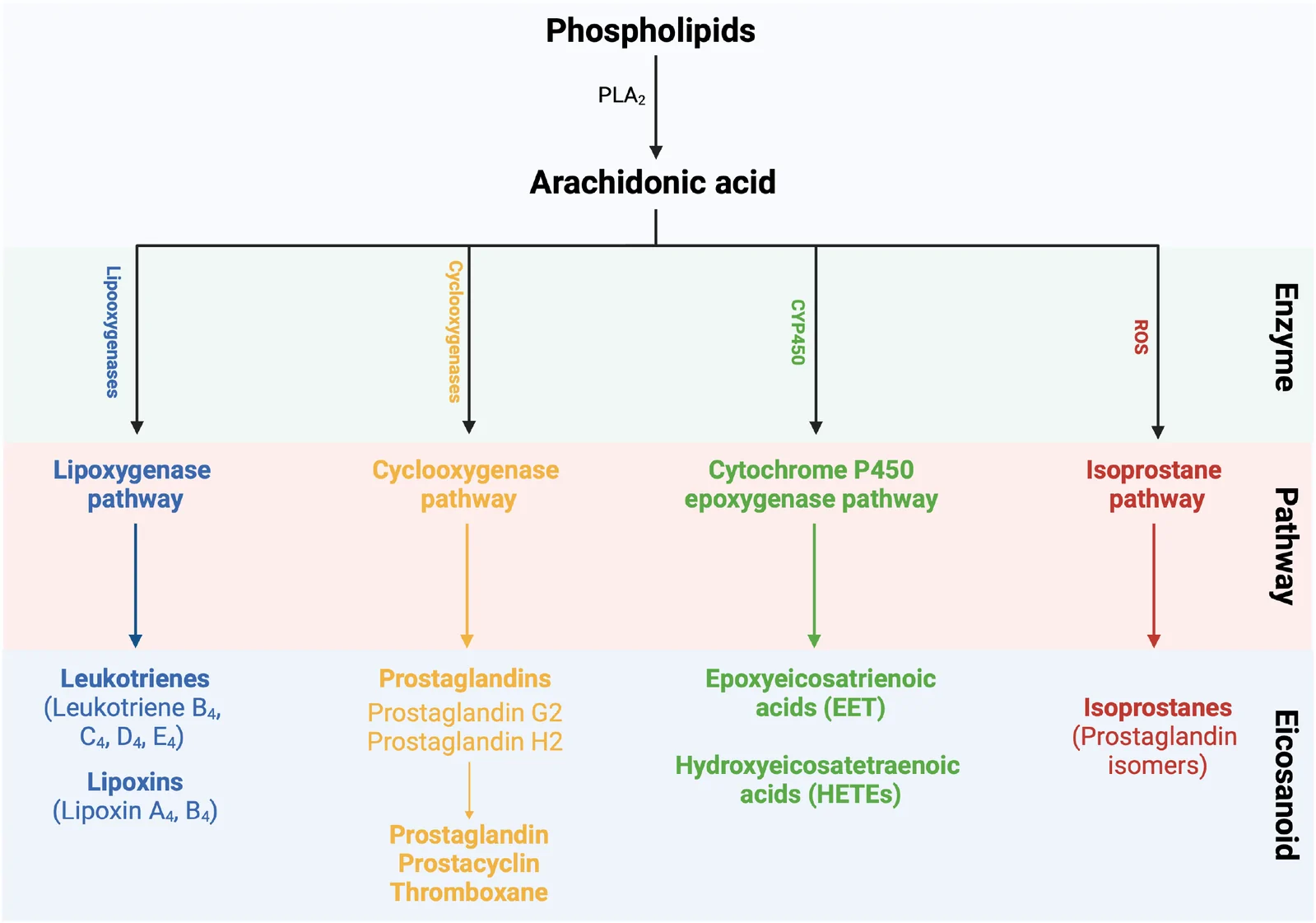
Enzymes and pathways involved in the production of eicosanoids from arachidonic acid. Metabolized through the cyclooxygenase (COX), lipoxygenase (LOX) and cytochrome P450 (cytP450) pathways, arachidonic acid (AA) is the substrate for eicosanoids that play unique roles in innate immune responses. The COX isoenzymes, catalyze the first step in the biosynthesis of prostaglandins (PG) which are among the most important mediators of inflammation (yellow text). The COX pathways form prostaglandins (PGs) and thromboxane (TXs) (yellow text) whereas lipoxygenase (LOX) pathways (blue text) form leukotrienes (LTs) and lipoxins (LXs) - all of which play significant roles in carcinogenesis. The CYTP450 pathways (green text) form various epoxy, hydroxy and dihydroxy derivatives that may exert protective effects in inflammatory disorders. As a non-enzymatic free radical-initiated peroxidation of AA, the isoprostane pathway (red text) produces a series of prostaglandin isomers.

The enzymatic processing of cell membrane phospholipids by phospholipases liberates lipid mediators that play key roles in membrane trafficking and signal transduction for proliferation and apoptosis. Several phospholipase families have been identified, including phospholipase A _1_ (PLA _1_), phospholipase A _2_ (PLA _2_), phospholipase C (PLC), and phospholipase D (PLD), each of which catalyzes the hydrolysis of phospholipids at distinct sites (6). However, the calcium (Ca^2+^)-dependent cytosolic phospholipase A_2_ (PLA_2_) is the only known phospholipase that possesses substrate specificity for arachidonic acid (AA), a 20-carbon, omega-6, polyunsaturated fatty acid (PUFA), which is esterified to form cell membrane phospholipids. **Figure 1** is an overview of the enzymes and pathways involved in the production of eicosanoids from phospholipids and AA.

PLA_2_ hydrolyses membrane phospholipids at the sn-2 position on the phospholipid backbone and releases free AA molecules into the cytoplasm in a single step (11). While many cells contain phospholipases, their expression, types, and level of activity differ depending on the specific cellular functions. Immune cells, such as macrophages and neutrophils, have higher levels of phospholipase activity, especially phospholipase A2 (PLA_2_), which plays a key role in the release of lipid mediators involved in inflammatory responses.

The activation of PLA_2_ occurs through various signaling pathways and stimuli including:

### Cell signaling and receptor activation

Many hormone receptors and neurotransmitters are coupled to cellular phospholipases and regulate the cytosolic levels of signaling molecules such as Ca^2+^, diacylglycerol, inositol trisphosphate, and AA.

### Ca2+ levels

An increase in cytosolic calcium levels activates Ca^2+^-dependent forms of PLA_2_ (12). Hormones like epinephrine, angiotensin, or platelet-derived growth factor also trigger PLA_2_ activation through cell surface receptors such as G-protein coupled receptors (GPCRs) or receptor tyrosine kinases (RTKs) (13).

### Inflammatory mediators

Cytokines, e.g., TNF-alpha and IL-1 activate PLA_2_. These substances are released during inflammatory immune responses and contribute to the production of pro-inflammatory lipid mediators (14).

### Oxidative stress

Reactive oxygen species (ROS) and antioxidants can activate PLA_2_ (15).

### Mechanical stress or injury

Physical damage or mechanical stress to the cell membrane triggers PLA_2_ activation (6).

## Cyclooxygenases

The two COX isoforms COX-1 and COX-2 catalyze the formation of thromboxane (TXA) and prostaglandins (PG) from AA (16). The gene encoding for COX-1 is considered a “housekeeping gene” due to low expression levels in most cell types. While COX-1 is constitutively expressed in many human tissues (16), the expression of COX-2 is induced by growth factors (17), cytokines (18), bacterial endotoxins (19) and tumor promoters (20). The function of COX-1 facilitates the production of prostaglandins and thromboxane, which play vital roles throughout the body. COX-1 is mainly found in mature blood platelets - the primary source of thromboxane A2​ that promotes clotting. The main effects of COX-2 are the production of prostaglandins that drive the inflammatory response. The production is initiated in response to cytokines released from damaged or infected cells (21). Both enzymes are major targets of non-steroidal anti-inflammatory drugs (NSAIDs) that prevent AA from reaching the enzyme’s catalytic pocket and thus inhibiting prostaglandin biosynthesis (22). A third cyclooxygenase isoform, termed COX-3, was initially described as a splice variant of the gene encoding COX-1. Although COX-3 has been identified in some species, subsequent studies have shown that the proposed human isoform is unlikely to encode a functional cyclooxygenase enzyme. Consequently, COX-3 is not generally regarded as a distinct functional cyclooxygenase in humans and has not been implicated in pancreatic ductal adenocarcinoma or other cancers to the same extent as COX-1 and COX-2 (23).

In some human cancers, COX-1 is markedly upregulated, e.g. in breast, ovarian, head and neck cancer, renal cell carcinoma, and hematological tumors^25.^ In colon cancer, COX-1 is required for the anchorage-independent growth ability of colon cancer cells, which is a key feature of the malignant phenotype (24). The upregulation of COX-1 in the tumor stroma is crucial in creating the chronic inflammation that promotes tumor growth, angiogenesis, and metastasis, among others by upregulating vascular endothelial growth factor (VEGF) and metalloproteinases (MMPs) (25, 26). Both COX-1 and COX-2 have been shown to specifically interact with the autoimmunity- and apoptosis-associated protein nucleobindin (Nuc). Nuc also plays a role in modulating calcium homeostasis in the cis-Golgi cisternae (27). Ballif and colleagues found that Nuc was extracellularly released when overexpressed on COX-1 cells. However, when Nuc was co-overexpressed with COX-1 or COX-2, its release was significantly reduced (28). This finding suggests that COX enzymes may be involved in the sequestration of Nuc within the lumen of the endoplasmatic reticulum (ER), where COX presumably controls the release of Nuc from the cell. Through this interaction, Nuc may also act as a potential regulator of COXs. The Nuc-COX complex could represent a prostaglandin-independent signaling mechanism for the chemopreventive activity of NSAIDs. In theory, NSAIDs could bind to COX and thereby cause changes in protein-protein interactions.

Inhibition of COX-1 significantly trigger tumor cell death by destroying the mitochondrial function associated with the blockage of the nuclear factor-κβ (NF-κB) signaling pathway (29). For some of these cancers, overexpression of COX-1 could represent a therapeutic target and a diagnostic biomarker (30).

COX-2 may be released to the tumor microenvironment (TME) by tumor cells and stromal cells, primarily cancer-associated fibroblasts (CAF), and tumor-associated macrophages (TAM) and it exerts most of its functions through its metabolite PGE_2_. Apart from driving inflammation, COX-2 promotes all aspects of tumor growth including cancer stem cell-like activity, apoptotic resistance, proliferation, angiogenesis, inflammation, invasion, and metastasis (31).

Multiple signals contribute to the effects of COX-2 on cancer cells with the mitogen-activated protein kinase (MAPK) family, epidermal growth factor (EGF), and NF-κB being the main upstream modulators in cancer cells.

Although the administration of COX-2 inhibitors in a preoperative setting in theory could reduce the risk of metastasis formation, COX-2-mediated intratumoral hypoxia and antiapoptotic activity favor resistance to chemotherapeutics. The cytotoxic therapies influence the inflammatory response and universally increase COX-2 mRNA levels in human cells. Also, the increased COX-2 and PGE_2_ levels derived from dying cancer cells are major barriers to antitumor immunity (32).

Early studies on prolonged use of COX-enzyme inhibitors, such as aspirin or other non-steroidal anti-inflammatory drugs (NSAIDs), show a significant reduction of colon cancer risk by 40-50% (33, 34). In a study with transgenic mice, overexpression of the COX-2 gene was found to promote breast cancer formation, and subsequent treatment with the selective COX-2 inhibitor celecoxib reduced tumor size (35).

A study by Masferrer et al. showed that COX-2 was expressed in the neo-vasculature of transplantable tumors in experimental animals (36). Inhibition of neovascularization was associated with decreased cell proliferation and increased apoptosis (36).

Furthermore, celecoxib significantly reduced the formation of premalignant polyps in the colon of patients with a high risk of colon cancer development (37). One study demonstrated a significantly reduced incidence of intestinal polyps in COX-2 knock-out mice with familial amyloid polyneuropathy (FAP) compared to COX-2 wild-type mice with FAP (38). This finding indicates that COX-2 may be involved in the formation of precursors for intestinal cancer in risk groups, such as patients with familial adenomatous polyposis (FAP) (38).

Studies have also revealed that the COX-2-associated changes contribute to the formation of the pre-metastatic niche (PMN) - a favorable microenvironment formed by pro-tumorigenic stromal cells and remodeling of the extracellular matrix receptive of circulating PDAC cells (39).

## Cyclooxygenases and prostaglandin E_2_

COX-1 expression is mostly absent or very low, and some cells also lack COX-2 expression which leaves them to rely on exogenous sources of prostaglandins derived from the TME, e.g. from fibroblasts and immune cells (40). Non-selective COX inhibitors, such as traditional NSAIDs, inhibit both COX-1 and COX-2 isoenzymes and are associated with gastrointestinal and renal adverse effects due to COX-1 inhibition in physiological tissues. In contrast, selective COX-2 inhibitors were developed to preferentially target the inducible COX-2 isoform, which is often upregulated in inflammatory conditions and cancer. This selectivity provides a more targeted approach to modulating prostaglandin E2 production within the tumor microenvironment while potentially reducing COX-1–related toxicity.

The PGH_2_ from AA is a precursor for important molecules associated with coagulation and inflammation, such as thromboxane A_2_ (TXA_2_) and PGE_2_. Particularly, PGE_2_ is of special interest in cancer biology as PG is highly expressed in numerous types of cancer (41, 42). Studies have revealed that PGE_2_ activates cancer-associated pathways and facilitates proliferation, invasion, and metastasis of cancer cells (41–43).

PGE_2_ has also been found to transactivate the epidermal growth factor receptor (EGFR) kinase cascade (44), which in turn can increase the biosynthesis of COX-2 (45), thereby enhancing the formation of PGE_2_ in a positive feedback loop.

One study evaluated the effectiveness of the selective COX-2 inhibitor apricoxib with and without standard therapy for PDAC (gemcitabine +/- erlotinib) in human PDAC cell lines (46). The study found apricoxib to reduce both tumor growth and metastasis formation in tumors with elevated COX-2 expression (46). Apricoxib reduced the IC_50_ for gemcitabine +/- erlotinib, enhanced the efficacy of the treatment, and reversed the epithelial-to-mesenchymal transition (EMT) (46).

Studies have also revealed that PGE_2_ can promote resistance to EGFR inhibitors, such as erlotinib, in non-small cell lung cancer (NSCLC) through facilitation of epithelial-to-mesenchymal transition (EMT) (47, 48). PGE_2_ may play a crucial role in tumorigenesis and resistance to anti-neoplastic treatments, presumably through the activation of pathways associated with EMT.

The elimination of PGE_2_ is initiated by two catabolic enzymes, 15-hydroxyprostaglandin dehydrogenase (15-PGDH) and 15-keto-prostaglandin-13-reductase (13-PGR). Interestingly, the expression of 15-PGDH has been reported to be reduced in human colorectal cancer (CRC), suggesting a link between tumorigenesis and high levels of PGE_2_ (49). Furthermore, low colonic 15-PGDH levels have been associated with resistance to celecoxib treatment in a colon adenoma prevention trial (50) indicating that appropriate 15-PGDH levels are needed for effective response to COX inhibitors.

Released by the FDA in 1999, celecoxib (Celebrex) and rofecoxib (Vioxx) were the first COX-2–selective NSAIDs available for clinical use. However, clinical trials with rheumatoid arthritis patients and chemoprevention in patients with colonic polyps (51) demonstrated increased cardiovascular risk with both drugs. That said, the doses used in the colon adenoma prevention trials were significantly higher than the typical doses used for pain relief. However, rofecoxib was withdrawn from the market in 2004. In contrast, celecoxib is still available and is one of the most commonly prescribed drugs in the US (11, 51).

Taken together, combining standard treatment for PDAC with COX inhibitors may enhance the effectiveness of anti-neoplastic therapies. However, adding celecoxib to gemcitabine in the treatment of PDAC has previously revealed mixed results (52, 53) as two different phase II trials evaluating the addition of the COX-2 inhibitor celecoxib to gemcitabine-based regimens in advanced pancreatic cancer demonstrated acceptable tolerability but no improvement in response rate or survival outcomes (54, 55). These findings suggest that celecoxib does not enhance the efficacy of gemcitabine or gemcitabine–cisplatin combinations. However, newer clinical trials are needed to further examine the potential of other combination approaches.

## COX-2 in pancreatic ductal adenocarcinoma

Clinical studies have demonstrated that COX-2 is upregulated in 42-90% of PDAC (56–59). Using immunohistochemical analysis, one study demonstrated COX-2 expression in 72% of invasive PDAC samples, while only 37% of the examined intraductal papillary-mucinous adenocarcinomas expressed COX-2 (60). Expression of COX-2 in PDAC was significantly associated with a poorer prognosis after tumor resection (10). The prognosis was particularly worse among patients with a tumor size ≥ 3 cm and overexpression of COX-2, suggesting that COX-2 influences the clinical outcome. The same study reported that COX-2 expression was not observed in the intraductal papillary-mucinous adenomas (10).

An *in vitro* study on PDAC cells co-cultured with fibroblasts showed an increased expression of the COX-2-gene in both tumor cells and stromal fibroblasts (61). Knocking out COX-2 via small interfering RNA and using the COX-2 selective inhibitor NS-398 resulted in a decrease in the invasiveness of the PDAC cells, emphasizing the potential involvement of COX-2 in tumor invasiveness and tumor progression (61).

One study examined the expression of COX-2 in ten pancreatic tumors using immunohistochemistry (IHC) and reverse transcription polymerase chain reaction (RT-PCR) (56). Compared to normal tissue, COX-2 expression was upregulated in 14 of 21 (67%) of the analyzed pancreatic tumors (59). Interestingly, stromal cells and infiltrating inflammatory cells of the TME did not express COX-2. For comparison, staining for COX-2 was minimal in normal pancreatic tissue (56). The absence of COX-2 expression in stromal cells is especially interesting as a study on human breast cancer tissue also located the expression of COX-2 in cancer cells, whereas the COX-1 expression was localized to the stroma of the cancer tissue alone (62). These findings indicate that COX-1 and COX-2 participate in the cellular and stromal changes associated with cancer, though acting on different compartments.

In five different PDAC cell lines, COX-2 mRNA levels were increased in four (BxPC-3, Capan-1, MDAPanc-3, MDAPanc-28) out of five (BxPC-3, Capan-1, MDA-Panc-3, MDA-Panc-28, PANC-1) (59). The NSAIDs, sulindac and NS-398, dose-dependently inhibited cell proliferation in all the analyzed cell lines, suggesting a possible anti-proliferative effect of COX inhibitors on PDAC cells (59).

Similar results were obtained in another independent study examining the COX-2 expression in PDAC tissue and PDAC cell lines (58). A wide range of COX-2 expression was reported in PDACs (0-93%), while the expression was much lower in the normal adjacent tissue (0-4%). In 56% of the analyzed PDAC specimens, COX-2 staining was positive, whereas all the non-tumorous tissue was COX-2-negative. COX-2 positivity or negativity were defined as COX-2 expression greater or less than 5%, respectively (58). Both mean and median COX-2 expression was significantly higher in the PDAC samples compared to normal pancreatic tissue (58). Through immunohistochemical staining, the COX-2 expression was then specifically localized to the cancer cells of the PDAC, while the expression was undetectable in the stroma of the same specimens. The same study investigated COX-2 expression in PDAC cell lines, and COX-2 activity was determined through measurement of PGE_2_ production (58). It was demonstrated that PGE_2_ production was increased in the COX-2-positive pancreatic tumor cell lines, whereas PGE_2_ levels in the COX-2-negative PDAC cell lines were minimal. Subsequent treatment of the COX-2-positive cell lines with the non-selective COX-inhibitor indomethacin and the COX-2 selective inhibitor NS-398, inhibited PGE_2_ production by 75% and 95%, respectively. Additionally, two cell lines, the highly COX-2-positive PDAC cell line BxPC-3 and the COX-2-negative PDAC cell line PaCa-2, were treated with COX-inhibitors sulindac, indomethacin and NS-398. All three COX inhibitors dose-dependently suppressed cell growth in both cell lines although the inhibition of cell growth was significantly greater in the COX-2-positive cell line (BxPC-3) treated with either indomethacin or NS-398 compared to the COX-2-negative cell line (PaCa-2) (58). Though the efficacy of COX-2 inhibitors seems more prominent in cancer cells expressing COX-2, these results suggest, that the effects of COX-2 inhibitors may not only be limited to COX-2-positive cancer cells.

The overexpression of COX-2 is usually correlated with a poor prognosis among patients with resectable PDAC (10). In a prospective cohort of 299 patients undergoing intended curative pancreaticoduodenectomy due to infiltrating PDAC, the COX-2 expression in tumor tissue was determined through immunohistochemistry, and survival was assessed by Kaplan-Meier survival analysis and multivariate Cox-analysis (10). The study reported a significantly worse prognosis among patients, whose PDAC expressed high levels of COX-2 (10). COX-2 expression was an independent prognostic factor for a worse survival with a hazard ratio (HR) of 1.41 (95% CI 1.08-1.84) when controlled for other prognostic PDAC factors.

A separate study examined the expression of COX-2 in human and hamster pancreatic neoplasia compared to adjacent normal pancreatic tissue (63). It demonstrated a significantly higher expression of COX-2 in pancreatic neoplasia in both hamster and human tissue compared to normal pancreatic tissue. The COX-2 expression increased significantly with neoplastic progression, except from stage PanIN2 to PanIN3 (63). The activity of COX-2 was estimated by PGE_2_ production, and a significantly greater average of the PGE_2_ protein was observed in PDAC compared to normal adjacent pancreatic tissue in both models, suggesting a correlation between COX-2 expression and COX-2 activity (63). The study also examined the effects of the non-selective COX inhibitor, sulindac, on tumor formation and tumor growth in hamsters. The formation of N-nitrosobis-(2-oxo- propyl)amine (BOP)-induced PDACs was reduced almost 5-fold by orally administrated sulindac. However, sulindac had little to no effect on tumor growth, indicating that sulindac and presumably other NSAIDs may be more potent at preventing tumor development. These results are similar to results from other studies examining COX chemoprevention in the colon (64). Further investigation is needed to illuminate the chemopreventive potential of COX inhibitors in PDAC.

## COX-2 and metastasis formation

In general, the formation of pre-metastatic niches (PMN) plays a key role in facilitating metastasis formation. The subject of PMN establishment has drawn great interest recently, though theories of PMN formation have long existed. One theory was proposed by the English surgeon Stephan Paget. Paget assumed that disseminating cancer cells (seeds) form metastases at locations where the microenvironment (soil) is most suitable (65). The theory is known as the “seed and soil theory” based on the findings that certain types of cancer prefer specific metastatic sites (65). The soil or the preselected metastatic site must undergo microenvironmental modulations to enhance the extravasation and growth of circulating cancer cells. To create the metastatic niche, tumor-secreted factors and tumor-derived exosomes rearrange the extracellular matrix and resident stromal cells to construct the perfect conditions for engraftment and growth of incoming tumor cells (66).

COX-2 and COX-2 derived PGE_2_ are found to be involved in the formation of PMN and consequently in the formation of metastases (67). For instance, mediastinal lymph node metastases were reduced by COX-2-inhibition in a murine lung cancer model, suggesting that COX-2 might be an important contributor to the early formation of metastases (39). Furthermore, COX-2-dependent PGE_2_ was reported to facilitate both angiogenesis and stromal tissue formation (68) and increase the activity of regulatory T-cells (Tregs), thus enhancing immunosuppression (69).

In PDAC, stromal fibroblasts increased the expression of genes related to invasion and metastasis (61). Particularly, the expression of COX-2, hyaluronic acid synthase-2 (HAS2) and matrix metalloproteinases (MMP) are upregulated by stromal fibroblasts in PDAC (61). MMPs are involved in the proteolytic degradation of the extracellular matrix (ECM) and cell migration (70). A study on PDAC cell lines found a positive correlation between mRNA levels of COX-2 and MMP-9 expression (71). Expression of MMP-9 was significantly downregulated in the COX-2-positive PDAC cell lines (BxPC-3 and Capan-1) treated with the selective COX-2 inhibitor NS398 (71). These findings suggest that COX-2 may promote invasion and metastasis through upregulation of MMP-9, thus facilitating cell migration. Similarly, hyaluronic acid is linked to cancer cell motility and enhanced metastasis (72). Increased production of hyaluronic acid in tumor cells was reported to trigger loss of E-cadherin and accumulation and nuclear translocation of cytoplasmic β-catenin in PDAC cells, consequently promoting EMT (73). COX-2 has been described as an important mediator of hyaluronic acid-induced effects (74). Hyaluronic acid induces COX-2 expression through interaction with CD44 (74). Inhibition of COX-2 leads to a decrease in hyaluronic acid production, furthering a reduction in COX-2 expression and suggesting a positive feedback loop between hyaluronic acid and COX-2 (74).

Also, COX-2 expression has been associated with angiogenesis through VEGF activation (75). A study analysis on PC-3 PDAC cell lines found a positive correlation between COX-2 expression and microvascular density (76). Administration of celecoxib to the PC-3 cell lines dose- and time-dependently inhibited PGE_2_ and VEGF formation, while exogenous administration of PGE_2_ helped maintain VEGF expression (76). These findings suggest that PGE_2_ can mediate both COX-2 and VEGF expression (76).

Moreover, the non-selective COX inhibitor acetylsalicylic acid (ASA) has been observed to induce additive anti-angiogenic effects of the VEGF-inhibitors sunitinib and DC101 by downregulation of VEGF-receptors and inhibition of VEGF secretion from thrombocytes (77).

COX-2-derived PGE_2_ inhibits differentiation/DNA binding 1 (Id-1) through activation of the MAPK signaling pathway in human glioblastoma cells (78). Id1 is a known functional marker for stem cell-like properties such as self-renewal and multipotency (79). Thus COX-2 might be involved in the upregulation of stem cell-like characteristics. Moreover, the Wnt/β-catenin signaling pathway is often overactive in different gastrointestinal cancers, including PDAC (80). Activation of β-catenin in colorectal cancer cells has been found to block the normal differentiation program of cells, thereby locking them in a progenitor-like state (43, 81) while overactive Wnt signaling is associated with cancer (82). In human CRC, cancer stem cells can be defined on the basis of high Wnt signaling activity, suggesting that the pathway influences the development of stem cell-like properties (83). Wnt has been linked to COX-2 and PGE_2_ in different studies (84, 85). Goessling and colleagues reported that Wnt activation of stem cells *in vivo* required PGE_2_ (85). A separate study found the Wnt/β-catenin signaling pathway to induce COX-2 expression in gastric cancer cell lines (86). The latter study also revealed that nuclear β-catenin levels correlated with COX-2 expression, suggesting a relation between COX-2 expression and Wnt/β-catenin signaling in gastric cancer (86). Likewise, in prostate cancer cell lines, expression of the Wnt/β-catenin signaling pathway was associated with tumor development and progression. The activity of the pathway could be blocked by celecoxib and the nitric oxide-donating aspirin derivate NO-ASA, resulting in decreased prostate cancer cell proliferation (87).

Wnt2, a member of the Wnt gene family, contributes to the migration and invasion of gastric cancer cells, resulting in cancer progression (88) as well as inducing tumor growth in esophageal cancer (88, 89). With PDAC, similar results have been obtained. Wnt2 is found to promote metastasis in PDAC (90), thereby contributing to a poorer prognosis. Patients whose pancreatic tumor had a high Wnt2 expression were found to have worse survival compared to patients whose tumor expressed little Wnt2 (90). In their univariate logistic regression analysis, Jiang et al. reported that patients with high Wnt2 expression had 2.712-times greater odds of lymph node metastasis than those with low expression (95% CI: 1.097–6.705) (90). These findings suggest that COX-2-activated Wnt/β-catenin signaling may contribute to tumor growth and progression, while COX inhibition may block the signaling pathway of Wnt/β-catenin.

In summary, COX-2 and its related pathways may contribute to changes in the tumor microenvironment that enhance cancer-associated properties such as invasion and metastasis formation.

## Immunosuppression through COX-2 related pathways

In carcinogenesis and cancer therapy, the role of the immune system has become a prominent research area. Especially, the immune checkpoint inhibitors such as programmed death ligand-1 (PD-L1) and cytotoxic T-lymphocyte-associated protein 4 (CTLA4) inhibitors, represent the auspicious leads for future cancer treatment.

The COX-2/microsomal prostaglandin E2-synthase1/PGE2 pathway is found to regulate the expression of PD-L1 in tumor-associated macrophages (TAMs) and myeloid-derived suppressor cells (MDSCs). A study found that co-culture of tumor cells with MDSCs stimulated PD-L1 expression, and bone-marrow derived cells (BMDCs) expressing PD-L1 were able to suppress the immune system by reducing the number of activated T-lymphocytes (91). BMDCs are a heterogeneous group of cells that originate in the bone marrow and are recruited to the tumor site by tumor secreted factors. Among others, BMDCs include immune cells like macrophages, neutrophils, MDSCs, and dendritic cells (92).

The tumor-infiltrating cells expression PD-L1 were observed to show high levels of COX-2 and microsomal PGE_2_ synthase 1 (mPGES1). When co-cultured BMDC and tumor cells were treated with COX-2 inhibitors and inhibitors of mPGES1, both the production of PGE_2_ and the expression of PD-L1 in myeloid cells were significantly reduced (91). Interestingly, the study also reported that the genetic expression of the PGE_2_ degrading enzyme 15-PGDH could influence PD-L1 expression, as high levels of 15-PGDH were found to reduce PD-L1 expression in tumor cells (91). It was concluded that the metabolism of PGE_2_ was involved in the regulation of PD-L1 expression on myeloid cells as well as tumor cells. Also, that tumor cells may affect the metabolism of PGE_2_ in MDSCs, consequently stimulating macrophages toward a PD-L1 positive differentiation and upregulating PD-L1 on tumor cells (91).

Increased COX-2 expression has also been linked to immunosuppression through COX-2-facilitated PGE_2_ production (93). The COX-2-PGE2 and proteinoid-receptor (COX-2-PGE_2_-EP) signaling pathway is found to suppress the activity of dendritic cells (DC) (94), T-cells (64, 95) and natural killer (NK)-cells (96, 97), while the same signaling-pathway promotes protumorigenic M2 macrophage differentiation (98). The M2 differentiation correlates with increased infiltration of CD4+ regulatory T cells (Tregs) and a reduced activity of CD8+ cytotoxic T-cells, both mechanisms which are known to suppress the adaptive immune system (97, 98).

Cytokines released by macrophages can stimulate the expression of COX-2 in tumor cells and other stromal cells. In addition to inhibiting COX enzyme activity, NSAIDs may suppress the expression of COX-2 and other proinflammatory gene products (99). Paik et al. reported that NSAIDs, flufenamic acid and sulindac sulfide, inhibited cytokine-mediated induction of COX-2 in HT-29 human colon cancer cells (99). This suppressive effect was mediated, in part, through inhibition of NF-κB. Using the macrophage-like cell line RAW 264.7. It was demonstrated that NSAIDs that inhibited NF-κB signaling also suppressed mitogen-induced expression of COX-2 and other proinflammatory molecules including iNOS and IL-1 (99).

High COX-2 expression is also associated with the expansion of MDSCs in different malignancies (100, 101). It is reported that MDSCs can inhibit T-cell differentiation and cytotoxicity, as well as reduce NK-cell activity through suppressive factors, such as nitric oxide synthase (NO-synthase), reactive oxygen species (ROS) and arginase-1 (102). MDSCs also recruit and expand Tregs through TGF-β and IL-10 production (103, 104). COX-2 has been found to maintain elevated levels of MDSC, thereby continually suppressing the activity of the adaptive immune system against malignant cells (93, 105). Treatment with the selective COX-2 inhibitor celecoxib reduced local and systemic expansion of MDSC (106) and improved the anti-neoplastic effects of immunotherapy (106). In a study on nasopharyngeal carcinomas, the expression of COX-2 correlated with the expansion of MDSC populations, and high COX-2 expression was found to promote metastasis (93, 107). COX-2-derived PGE_2_ contributes to an immunosuppressive tumor microenvironment by impairing dendritic cell maturation, promoting regulatory T cell activity, and limiting cytotoxic T cell infiltration (108, 109). These mechanisms may reduce the efficacy of immune checkpoint inhibitors such as anti-PD-1/PD-L1 therapies. Consequently, COX-2 inhibition has been proposed as a strategy to reprogram the tumor microenvironment and enhance responsiveness to immune checkpoint blockade.

In summary, preclinical evidence suggests that targeting COX-2/PGE2 signaling may improve T cell infiltration and synergize with checkpoint inhibition, although clinical validation in PDAC remains limited and warrants further investigation.

## Selective COX inhibitors also act through COX-independent mechanisms

Although convincing evidence indicates that NSAIDs may exert their antitumor effects through COX-2 inhibition, the molecular mechanisms of their chemopreventive and chemotherapeutic actions remain largely unknown. Investigators postulate that the antitumor effects of NSAIDs may not entirely result from inhibition of COX, but that other cellular targets likely contribute to the chemopreventive activity of these agents. Supporting this hypothesis is the observation that high concentrations of NSAIDs inhibit the growth of cancer cell lines that do not express either COX-1 or COX-2 (110–112).

Weinstein and colleagues found that treating cancer cell lines as well as experimental animals with sulindac sulfone, a metabolite of the NSAID sulindac sulfoxide, inhibited growth and apoptosis without inhibiting COX (113). In patients with a history of prostate cancer, sulindac sulfone treatment resulted in a significant decrease in prostate-specific antigen (PSA) levels compared with placebo (114). Mechanistic studies uncovered that sulindac sulfone and related derivatives promoted apoptosis via inhibition of cGMP-specific phosphodiesterases PDE_2_ and PDE_5_, resulting in increased levels of cGMP and activation of protein kinase G (115). Activation of protein kinase G, in turn, induces apoptosis, partially through activation of JNK1, a protein kinase of the MAPK family (116).

Certain NSAIDs block the activation of NF-κB. The NF-κB transcription factors regulate the expression of several genes involved in inflammation and carcinogenesis. Aspirin and salicylate have been reported to inhibit the activity of IKK_β_, thereby preventing I_κ_B_α_ phosphorylation and NF-κB activation (117). Furthermore, sulindac sulfide inhibited both IKK_α_ and IKK_β_ kinase activity and promoted apoptosis in the colon cancer cell line HCT-15, which lacks COX-2. By contrast, neither indomethacin nor ibuprofen inhibited IKK kinase activity. These findings suggest that certain NSAIDs may not only act by inhibiting COXs but may also target the NF-κB pathway.

NSAIDs have been reported to inhibit the expression of the antiapoptotic protein, Bcl-X_L_, resulting in a significant increase in the BAX: Bcl-X_L_ ratio and subsequent mitochondrial-mediated apoptosis (118). Interestingly, PPAR*δ* was revealed to be a target of both NSAIDs and tumor suppressor protein, adenomatous polyposis coli (APC), in human colorectal cancer cells (119). Normally, APC downregulates the expression of PPAR*δ*, however, in colorectal cancer, APC mutations are common and as a consequence, increased amounts of PPAR*δ* occur at a high frequency. Treating the cells with NSAIDs, such as sulindac sulfide and indomethacin, partially compensated for an APC mutation by disrupting the DNA binding activity of PPAR*δ*. On the other hand, overexpression of PPAR*δ* rescued, in part, colorectal cancer cells from sulindac sulfide-induced apoptosis. Additional experiments confirmed that genetic disruption of PPAR*δ* did not alter the sensitivity of the cells to NSAIDs (120). Furthermore, when inoculated as xenografts in nude mice, PPAR*δ* ^-/-^ cells exhibited a decreased ability to form tumors compared with PPAR*δ* ^+/-^ and wild-type controls (120). In patients with PDAC, PPAR*δ* levels correlate with advanced pathological tumor stage, increased risk of tumor recurrence and distant metastases (121). Taken together, these results suggest that NSAID-mediated inhibition of PPAR*δ* might represent a therapeutic strategy for cancer.

**Table 1** summarizes the discussed preclinical studies on COX expression and inhibition in pancreatic cancer. **Table 2** summarizes results from clinical studies on COX expression and inhibition.

**Table 1:** Preclinical studies on COX in pancreatic ductal adenocarcinomas.

Study	Model	Main finding	Therapeutic implication
Tucker et al.*	PDAC cell lines and tissue	COX-2 mRNA upregulated in all examined PDAC samples. Minimal expression in normal pancreas.	Supports COX-2 as a biomarker and therapeutic target.
Molina et al.*	PDAC cell lines and tissue	NSAIDs (sulindac, NS-398) inhibited proliferation of PDAC cells.	COX inhibition may suppress tumour growth.
Yip-Schneider et al.*	PDAC cell lines and tissue	COX-2 expression associated with increased PGE2 production. COX inhibitors reduced proliferation.	COX-2/PGE2 pathway contributes to tumour progression.
Koshiba et al.*	PDAC cell lines and tissue	COX-2 expression in PDAC	COX-2 expressed in 72% of invasive PDACs
Tucker et al.*	PDAC cell lines and tissue	COX-2 expression in PDAC	COX-2 strongly upregulated compared with normal pancreas
Sato et al.	PDAC cells and stromal fibroblast co-culture	Stromal fibroblasts increased COX-2 expression and invasiveness.	Tumour-stroma interactions promote metastasis.
Crowell et al.*	Animal PDAC model	Sulindac reduced PDAC formation nearly five-fold.	Possible chemopreventive role of NSAIDs.
Kirane et al.	Human PDAC xenograft models	Apricoxib reduced tumour growth, metastasis, and EMT while improving gemcitabine response.	Combination therapy may enhance chemotherapy efficacy.
Bu et al.	PDAC cell lines	COX-2 inhibition reduced MMP-9 expression.	COX-2 may facilitate invasion and metastasis.
Xie et al.*	PDAC cell lines	Celecoxib reduced VEGF and angiogenesis via PGE2 inhibition.	COX-2 contributes to tumour angiogenesis.
Hill et al.	Mouse PDAC model	COX-2 activated PI3K/AKT signalling during tumour development.	Cell-intrinsic oncogenic role of COX-2.

* Includes both preclinical data and human tissue analysis. COX, Cyclooxygenase; NSAIDs, non-steroidal anti-inflammatory drugs; PDAC, pancreatic ductal adenocarcinoma, PGE2, prostaglandin E2; VEGF, vascular endothelial growth factor.

**Table 2:** Clinical studies evaluating COX-2 expression and COX inhibition.

Study	Study design	Intervention / focus	Main findings
Matsubayashi et al.	Prospective cohort	COX-2 expression in PDAC	High COX-2 expression associated with worse survival (HR 1.41)
Dragovich et al.	Phase II trial	Gemcitabine + celecoxib	Acceptable tolerability but no survival benefit
El-Rayes et al.	Phase II trial	Gemcitabine/cisplatin + celecoxib	No improvement in response rate or survival
Solomon et al.	Clinical safety study	Celecoxib	Increased cardiovascular risk with prolonged/high-dose COX-2 inhibition
ALASCCA trial	Randomized clinical trial	Adjuvant aspirin	Reduced recurrence risk in PIK3CA-mutated colorectal tumours; supports future investigation in PDAC

COX, Cyclooxygenase; NSAIDs, non-steroidal anti-inflammatory drugs; PDAC, pancreatic ductal adenocarcinoma..

## Discussion

Increased amounts of COX-2 are detected in numerous solid tumors including PDAC. Studies suggest that overexpression of COX-2 is a consequence of increased transcription and enhanced stability of COX-2 mRNA. Further evidence finds a correlation between elevated COX-2 expression in various human cancers and outcome, highlighting the importance of COX-2 as a target for therapy.

DuBois and colleagues showed that overexpression of COX-2 in intestinal epithelial cells inhibited apoptosis and stimulated angiogenesis (122). The group found that treatment with a selective COX-2 inhibitor suppressed the growth of experimental colorectal carcinoma without detrimental effects to the normal intestine (123). Celecoxib, a selective COX-2 inhibitor, has been shown to reduce tumor growth and metastasis in xenograft tumor models (36). The antineoplastic activity of celecoxib is thought to be due in part to inhibition of angiogenesis (122). Supporting this idea is the observation that prostaglandins stimulate the production of vascular endothelial growth factor (VEGF) (124).

Although the anti-neoplastic effects of COX-inhibitors have been established, some of the molecular mechanisms remain elusive. Oncogenes enhance the expression of COX-2, but interactions between tumor suppressor genes and COX-2 have been scarcely studied. It has been shown that the transduction of cancer cells with p53 tumor suppressor gene decreases the synthesis of VEGF and inhibits tumor cell-induced angiogenesis (125). In a study with mouse embryo fibroblasts, the levels of COX-2 protein and mRNA were significantly suppressed by wild-type p53 but not by mutant p53. Cells expressing wild-type p53 showed about a 10-fold decrease in synthesis of PGE_2_ compared with those expressing mutant p53 (126). Interactions between p53 and COX-2 could explain why levels of COX-2 are undetectable in normal epithelial cells and elevated in many cancers. Mutations of p53 may, in other words, contribute to the increased expression of Cox-2 that is observed in malignant tissues.

COX-2 and PGE_2_-associated pathways are involved in the facilitation of angiogenesis, immunosuppression, epithelial-to-mesenchymal transition, cellular migration, stem cell-like properties, invasion and metastasis, which are all known hallmarks of cancer (127). The COX-2-mediated changes in PDAC are strikingly similar to those reported in other cancers such as colorectal, gastric, esophageal and prostate cancer, suggesting that COX-2 may play an important function in cancer formation and progression in general.

Though most of the studies examining the effects of COX inhibitors on cancer uses selective COX-2 inhibitors, the effects of non-selective COX inhibitors could reveal even more promising results as COX-2 is overexpressed in cancer cells, whereas COX-1 is found to be overexpressed in the cancer stroma (62). Furthermore, since COX-1 is expressed constitutively in most tissues, knocking out the COX-2 gene or selectively inhibiting COX-2 is not expected to completely block the synthesis of prostanoids in tumor tissues. However, data on the importance of COX-1 are scarce and further research is warranted and targeting both COX enzymes at the same time with a non-selective and irreversible COX inhibitor (acetylic salicylic acid) could represent more effective approach and result in superior outcomes than using selective COX-2 inhibitors.

The combination of COX inhibitors with other growth-inhibiting agents has been studied by other groups, e.g. Peulen and colleagues, who examined the anti-tumorigenic effects of class I histone deacetylase (HDAC) inhibition in combination with selective COX-2 inhibitors on human PDAC cell lines (128). Inhibition of HDAC and COX-2 in combination, significantly impaired cancer cell growth in the pancreas cancer cell lines through the NF-κB pathway (128). The team discussed the benefits of combining COX-2 inhibitors with HDAC inhibitors as an additional treatment for optimizing the standard anti-neoplastic treatment strategies against PDAC (128).

More recently, a meta-analysis investigating the efficacy of COX-2-inhibition combined with chemotherapy for non-small-cell lung cancer (NSCLC) found COX-2 inhibitors demonstrated an improved overall response rate to chemotherapy among treated patients (129). However, the addition of COX-2 inhibitors did not increase survival rates, and the authors pointed to an accentuated risk of cardiovascular events related to the use of COX-2 inhibitors (129).

Although this review offers contextual insight and identifies key trends in research on COX inhibitors in PDAC, its narrative nature represents an inherent limitation, as the included literature was not identified through a systematic selection process. This may introduce bias in the choice and interpretation of the discussed studies. Another limitation is that several of the discussed and presented preclinical studies in this paper were conducted on xenograft models. While xenograft models are widely used in preclinical research, their lack of a functional immune system limits their ability to capture immune-mediated processes such as COX-2/PGE2 signaling, and syngeneic or chemically induced models may therefore offer more physiologically relevant insight into tumor–immune interactions.

## Future perspectives

The histopathological analyses of COX-1 and COX-2 expression in cancer tissue could be of particular significance for optimizing the effects of anti-neoplastic therapies with COX inhibitors, especially in PDAC as treatment opportunities are scarce. Studies have revealed that the expression of COX-2 in cancer cells determines the effectiveness of treatment with COX inhibitors. Therefore, COX inhibitors could be specifically relevant among patients with COX-2-positive tumors. Since local levels of the COX-2 degrading enzyme, 15-PGDH, were found to influence the responsiveness to treatment with COX inhibitors in colon cancer, it should be investigated if 15-PGDH levels are linked to an improved COX inhibitor response in PDAC.

Beyond conventional NSAIDs and selective COX-2 inhibitors, novel drug design strategies, including NSAID-metal complexes and NSAID conjugates incorporating mitochondria-targeting or other antiproliferative moieties, are being explored to enhance antitumor efficacy while reducing systemic toxicity. Although these approaches have shown promising preclinical activity (130, 131), evidence in PDAC remains limited, and their clinical relevance has yet to be established.

The clinical translation of COX-targeted therapies in PDAC remains challenging. One of the major obstacles is the lack of validated predictive biomarkers to identify patients most likely to benefit from COX inhibition. While elevated COX-2 expression has been associated with poor prognosis and may represent a potential biomarker, additional molecular and inflammatory characteristics, including prostaglandin pathway activity, immune cell infiltration, and tumor-specific transcriptional profiles, are likely to influence treatment response and require prospective validation. Furthermore, increasing evidence suggests that COX inhibitors may be most effective when incorporated into combination treatment strategies. Future clinical trials incorporating biomarker-guided patient stratification together with rational combination therapies will be essential to define the therapeutic role of COX-targeted approaches in PDAC. Newly published data from the ALASCCA trial support the biomarker-guided patient stratification strategy (132). In patients with localized colorectal cancer harboring PI3K pathway alterations, adjuvant treatment with low-dose aspirin (160 mg daily for 3 years) significantly reduced the risk of cancer recurrence, particularly in patients with PIK3CA hotspot mutations (HR = 0.49) (132). Improvements in disease-free survival were also observed, with an acceptable safety profile and only a modest increase in adverse events compared with placebo (132). As the only non-selective, irreversible COX inhibitor, aspirin has attracted particular attention in cancer treatment, both as a preventive agent and in combination with chemotherapy.

To our knowledge, there have not been conducted clinical trials examining the potency and safety of solely on aspirin as an add-on to standard chemotherapy or immunotherapy for PDAC. Prolonged inhibition of COX-2 is associated with cardiovascular, as well as gastrointestinal side effects (51, 133, 134), which should be considered especially when planning future clinical trials. Despite their therapeutic potential, the long-term use of selective COX-2 inhibitors is limited by concerns regarding cardiovascular safety. Selective inhibition of COX-2 reduces endothelial prostacyclin (PGI_2_) production while largely preserving platelet-derived TXA_2_ synthesis mediated by COX-1, thereby shifting the hemostatic balance toward a prothrombotic state. This imbalance has been associated with an increased risk of adverse cardiovascular events, including myocardial infarction and stroke, particularly with prolonged treatment or in patients with pre-existing cardiovascular disease (135, 136). Consequently, the potential benefits of COX-2 inhibition in PDAC must be carefully weighed against these risks, and future therapeutic strategies may require biomarker-guided patient selection, optimized dosing regimens, or combination approaches that maximize antitumor efficacy while minimizing systemic toxicity. While COX inhibition directly and indirectly alters several signal transduction pathways that are involved in cancer, the studies discussed in this narrative review primarily investigated the efficacy of COX inhibitors on cancer-associated changes in PDAC. However, studies examining the efficacy and safety of combining anti-neoplastic treatment regimens with COX inhibitors for the treatment of PDAC are sparse and further clinical studies are demanded.

In summary, the available preclinical evidence is derived predominantly from established PDAC cell lines and xenograft mouse models, with selective COX-2 inhibitors, particularly celecoxib, being the most extensively investigated therapeutic agents. Although most studies consistently report reduced tumor growth, proliferation, and metastatic potential following COX inhibition, considerable heterogeneity exists with respect to experimental models, treatment regimens, and outcome measures. These differences, together with the limited number of well-powered clinical studies, underscore the need for standardized preclinical approaches and biomarker-driven clinical trials to better define the role of COX-targeted therapies in PDAC.

## Conclusion

The review concludes that COX-2 and its derived prostaglandins play a fundamental role in the aggressive nature of PDAC by both driving intrinsic tumor growth and by shielding the tumor from the host immune response. Although PDAC remains one of the most lethal malignancies with a 5-year survival rate below 10%, the clinical evidence suggests that targeted inhibition of the COX-2 axis represents a promising therapeutic strategy. By combining COX inhibitors with modern immunotherapy or standard chemotherapy, it may be possible to disrupt the tumor’s protective barrier and significantly improve patient prognosis. Currently, clinical studies examining COX inhibitors in combination with anti-neoplastic therapies are sparse, emphasizing the necessity for further research on the matter, particularly in the treatment of PDAC.

## References

[1] Siegel RL Miller KD Jemal A . Cancer statistics, 2018. CA Cancer J Clin. (2018) 68:7–30. doi: 10.3322/caac.21442 29313949

[2] McGuigan A Kelly P Turkington R Jones C Coleman H McCain RS . Pancreatic cancer: a review of clinical diagnosis, epidemiology, treatment and outcomes. World J Gastroenterol. (2018) 24:4846–61. doi: 10.3748/wjg.v24.i43.4846 30487695 PMC6250924

[3] Saad AM Turk T Al-Husseini MJ Abdel-Rahman O . Trends in pancreatic adenocarcinoma incidence and mortality in the United States in the last four decades; a SEER-based study. BMC Cancer. (2018) 18:688. doi: 10.1186/s12885-018-4610-4 29940910 PMC6020186

[4] Mantovani A Allavena P Sica A Balkwill F . Cancer-related inflammation. Nat 2008 454:7203. (2008) 454:436–44. doi: 10.1016/b978-0-12-801238-3.65801-4 18650914

[5] Hanahan D . Hallmarks of cancer: new dimensions. Cancer Discov. (2022) 12:31–46. doi: 10.1158/2159-8290.cd-21-1059 35022204

[6] Dennis EA Norris PC . Eicosanoid storm in infection and inflammation. Nat Rev Immunol 2015 15:8. (2015) 15:511–23. doi: 10.1038/nri3859 26139350 PMC4606863

[7] Krishnamachary B Stasinopoulos I Kakkad S Penet M-F Jacob D Wildes F . Breast cancer cell cyclooxygenase-2 expression alters extracellular matrix structure and function and numbers of cancer associated fibroblasts. Oncotarget. (2017) 8:17981–94. doi: 10.18632/oncotarget.14912 28152501 PMC5392301

[8] Kirschenbaum A Liu X-H Yao S Levine AC . The role of cyclooxygenase-2 in prostate cancer. Urology. (2001) 58:127–31. doi: 10.1016/s0090-4295(01)01255-9 11502467

[9] Petkova DK Clelland C Ronan J Pang L Coulson JM Lewis S . Overexpression of cyclooxygenase-2 in non-small cell lung cancer. Respir Med. (2004) 98:164–72. doi: 10.1016/j.rmed.2003.09.006 14971881

[10] Matsubayashi H Infante J Winter J Klein A Schulick R Hruban R . Tumor COX-2 expression and prognosis of patients with resectable pancreatic cancer. Cancer Biol Ther. (2007) 6:1569–75. doi: 10.4161/cbt.6.10.4711 18000398

[11] Dennis EA Cao J Hsu YH Magrioti V Kokotos G . Phospholipase A2 enzymes: physical structure, biological function, disease implication, chemical inhibition, and therapeutic intervention. Chem Rev. (2011) 111:6130–85. doi: 10.1016/b0-12-443710-9/00478-6 21910409 PMC3196595

[12] Khan SA Ilies MA . The phospholipase A2 superfamily: structure, isozymes, catalysis, physiologic and pathologic roles. Int J Mol Sci. (2023) 24:1353. doi: 10.3390/ijms24021353 36674864 PMC9862071

[13] Souza JL Martins-Cardoso K Guimarães IS de Melo AC Lopes AH Monteiro RQ . Interplay between EGFR and the platelet-activating factor/PAF receptor signaling axis mediates aggressive behavior of cervical cancer. Front Oncol. (2020) 10:557280. doi: 10.3389/fonc.2020.557280 33392068 PMC7773908

[14] Lin CC Lin WN Cho RL Wang C Hsiao LD Yang CM . TNF-α-induced cPLA2 expression via NADPH oxidase/reactive oxygen species-dependent NF-κB cascade on human pulmonary alveolar epithelial cells. Front Pharmacol. (2016) 7:222319. doi: 10.3389/fphar.2016.00447 27932980 PMC5122718

[15] Chuang DY Simonyi A Kotzbauer PT Gu Z Sun GY . Cytosolic phospholipase A2 plays a crucial role in ROS/NO signaling during microglial activation through the lipoxygenase pathway. J Neuroinflamm. (2015) 12. doi: 10.1186/S12974-015-0419-0 26520095 PMC4628268

[16] Crofford L . COX-1 and COX-2 tissue expression: implications and predictions. J Rheumatol Suppl. (1997) 49:15–9. 9249646

[17] Leung WK To KF Ng YP Lee TL Lau JY Chan FK . Association between cyclo-oxygenase-2 overexpression and missense p53 mutations in gastric cancer. Br J Cancer. (2001) 84:335–9. doi: 10.1054/bjoc.2000.1607 11161397 PMC2363738

[18] Matsuura H Sakaue M Subbaramaiah K Kamitani H Eling TE Dannenberg AJ . Regulation of cyclooxygenase-2 by interferon γ and transforming growth factor α in normal human epidermal keratinocytes and squamous carcinoma cells: role of mitogen-activated protein kinases. J Biol Chem. (1999) 274:29138–48. doi: 10.1074/jbc.274.41.29138 10506169

[19] Kirkby NS Zaiss AK Wright WR Jiao J Chan MV Warner TD . Differential COX-2 induction by viral and bacterial PAMPs: consequences for cytokine and interferon responses and implications for anti-viral COX-2 directed therapies. Biochem Biophys Res Commun. (2013) 438:249–56. doi: 10.1016/j.bbrc.2013.07.006 23850620 PMC3759847

[20] Inoue H Umesono K Nishimori T Hirata Y Tanabe T . Glucocorticoid-mediated suppression of the promoter activity of the cyclooxygenase-2 gene is modulated by expression of its receptor in vascular endothelial cells. Biochem Biophys Res Commun. (1999) 254:292–8. doi: 10.1006/bbrc.1998.9939 9918831

[21] Allaj V Guo C Nie D . Non-steroid anti-inflammatory drugs, prostaglandins, and cancer. Cell Bioscience 2013 3:1. (2013) 3:8. doi: 10.1186/2045-3701-3-8 23388178 PMC3599181

[22] Miciaccia M Belviso BD Iaselli M Cingolani G Ferorelli S Cappellari M . Three-dimensional structure of human cyclooxygenase (hCOX)-1. Sci Rep. (2021) 11:1–18. doi: 10.1038/s41598-021-83438-z 33619313 PMC7900114

[23] Warner TD Mitchell JA . Cyclooxygenase-3 (COX-3): filling in the gaps toward a COX continuum? Proc Natl Acad Sci USA. (2002) 99:13371–3. doi: 10.1073/pnas.222543099 12374850 PMC129677

[24] Li H Zhu F Chen H Cheng KW Zykova T Oi N . 6-C-(E-phenylethenyl)-naringenin suppresses colorectal cancer growth by inhibiting cyclooxygenase-1. Cancer Res. (2014) 74:243–52. doi: 10.1158/1538-7445.am2014-1252 24220240 PMC3947334

[25] Wilson AJ Fadare O Beeghly-Fadiel A Son DS Liu Q Zhao S . Aberrant over-expression of COX-1 intersects multiple pro-tumorigenic pathways in high-grade serous ovarian cancer. Oncotarget. (2015) 6:21353–68. doi: 10.18632/oncotarget.3860 25972361 PMC4673270

[26] Osman WM Youssef NS . Combined use of COX-1 and VEGF immunohistochemistry refines the histopathologic prognosis of renal cell carcinoma. Int J Clin Exp Pathol. (2015) 8:8165. doi: 10.53347/rid-22630 26339385 PMC4555713

[27] Lin P Le-Niculescu H Hofmeister R McCaffery JM Jin M Hennemann H . The mammalian calcium-binding protein, nucleobindin (CALNUC), is a Golgi resident protein. J Cell Biol. (1998) 141:1515–27. doi: 10.1083/jcb.141.7.1515 9647645 PMC2132997

[28] Ballif BA Mincek NV Barratt JT Wilson ML Simmons DL . Interaction of cyclooxygenases with an apoptosis- and autoimmunity-associated protein. Proc Natl Acad Sci. (1996) 93:5544–9. doi: 10.1073/pnas.93.11.5544 8643612 PMC39283

[29] Ding L Gu H Lan Z Lei Q Wang W Ruan J . Downregulation of cyclooxygenase-1 stimulates mitochondrial apoptosis through the NF-κB signaling pathway in colorectal cancer cells. Oncol Rep. (2019) 41:559–69. doi: 10.3892/or.2018.6785 30320345

[30] Perrone MG Malerba P Uddin MJ Vitale P Panella A Crews BC . PET radiotracer [^18^F]-P6 selectively targeting COX-1 as a novel biomarker in ovarian cancer: preliminary investigation. Eur J Med Chem. (2014) 80:562–8. doi: 10.3998/ark.5550190.0007.810 PMC4401082 24832612

[31] Hashemi Goradel N Najafi M Salehi E Farhood B Mortezaee K . Cyclooxygenase-2 in cancer: a review. J Cell Physiol. (2019) 234:5683–99. doi: 10.1002/jcp.27411 30341914

[32] Bell CR Pelly VS Moeini A Chiang SC Flanagan E Bromley CP . Chemotherapy-induced COX-2 upregulation by cancer cells defines their inflammatory properties and limits the efficacy of chemoimmunotherapy combinations. Nat Commun. (2022) 13. doi: 10.1038/S41467-022-29606-9 35440553 PMC9018752

[33] Kune GA Kune S Watson LF . Colorectal cancer risk, chronic illnesses, operations, and medications: case control results from the Melbourne Colorectal Cancer Study. Cancer Res. (1988) 48:4399–404. doi: 10.1007/bf02554806 3390835

[34] Thun MJ Namboodiri MM Heath CW . Aspirin use and reduced risk of fatal colon cancer. N Engl J Med. (1991) 325:1593–6. doi: 10.1056/nejm199112053252301 1669840

[35] Narko K Zweifel B Trifan O Ristimäki A Lane TF Hla T . COX-2 inhibitors and genetic background reduce mammary tumorigenesis in cyclooxygenase-2 transgenic mice. Prostaglandins Other Lipid Mediat. (2005) 76:86–94. doi: 10.1016/j.prostaglandins.2005.01.002 15967164

[36] Masferrer J Leahy K Koki A Zweifel BS Settle S Woerner BM . Antiangiogenic and antitumor activities of cyclooxygenase-2 inhibitors. Cancer Res. (2000) 60:1306–11. doi: 10.1016/s0889-8553(05)70252-1 10728691

[37] Bertagnolli MM Eagle CJ Zauber AG Redston M Solomon SD Kim K . Celecoxib for the prevention of sporadic colorectal adenomas. N Engl J Med. (2006) 355:873–84. doi: 10.1056/nejmoa061355 16943400

[38] Oshima M Dinchuk JE Kargman SL Oshima H Hancock B Kwong E . Suppression of intestinal polyposis in ApcΔ716 knockout mice by inhibition of cyclooxygenase 2 (COX-2). Cell. (1996) 87:803–9. doi: 10.1016/s0092-8674(00)81988-1 8945508

[39] Ogawa F Amano H Eshima K Ito Y Matsui Y Hosono K . Prostanoid induces premetastatic niche in regional lymph nodes. J Clin Invest. (2014) 124:4882–94. doi: 10.1172/jci73530 25271626 PMC4347225

[40] Omura N Griffith M Vincent A Li A Hong SM Walter K . Cyclooxygenase-deficient pancreatic cancer cells utilize exogenous sources of prostaglandins. Mol Cancer Res. (2010) 8:821. doi: 10.1158/1541-7786.mcr-09-0336 20530583 PMC2888921

[41] Maeng H-J Lee W-J Jin Q-R Chang J-E Shim W-S . Upregulation of COX-2 in the lung cancer promotes overexpression of multidrug resistance protein 4 (MRP4) via PGE2-dependent pathway. Eur J Pharm Sci. (2014) 62:189–96. doi: 10.1016/j.ejps.2014.05.023 24909729

[42] Wang D Dubois RN . Prostaglandins and cancer. Gut. (2006) 55:115–22. doi: 10.1136/gut.2004.047100 16118353 PMC1856377

[43] Greenhough A Moore AE Williams AC Roberts HR Smartt HJM Paraskeva C . The COX-2/PGE 2 pathway: key roles in the hallmarks of cancer and adaptation to the tumour microenvironment. Carcinogenesis. (2009) 30:377–86. doi: 10.1093/carcin/bgp014 19136477

[44] Pai R Soreghan B Szabo IL Pavelka M Baatar D Tarnawski AS . Prostaglandin E2 transactivates EGF receptor: a novel mechanism for promoting colon cancer growth and gastrointestinal hypertrophy. Nat Med. (2002) 8:289. doi: 10.1038/nm0302-289 11875501

[45] Coffey RJ Hawkey CJ Damstrup L Graves-Deal R Daniel VC Dempsey PJ . Epidermal growth factor receptor activation induces nuclear targeting of cyclooxygenase-2, basolateral release of prostaglandins, and mitogenesis in polarizing colon cancer cells. Proc Natl Acad Sci USA. (1997) 94:657–62. doi: 10.1073/pnas.94.2.657 9012840 PMC19569

[46] Kirane A Toombs JE Ostapoff K Carbon JG Zaknoen S Braunfeld J . Apricoxib, a novel inhibitor of COX-2, markedly improves standard therapy response in molecularly defined models of pancreatic cancer. Clin Cancer Res. (2012) 18:5031–42. doi: 10.1158/1078-0432.ccr-12-0453 22829202 PMC3777527

[47] Reckamp K Gitlitz B Chen LC Patel R Milne G Syto M . Biomarker-based phase I dose-escalation, pharmacokinetic, and pharmacodynamic study of oral apricoxib in combination with erlotinib in advanced nonsmall cell lung cancer. Cancer. (2011) 117:809–18. doi: 10.1002/cncr.25473 20922800

[48] Kirane A Toombs JE Ostapoff KT Brekken RA Larsen JE Meshaw KR . Epithelial–mesenchymal transition increases tumor sensitivity to COX-2 inhibition by apricoxib. Carcinogenesis. (2012) 33:1639–46. doi: 10.1093/carcin/bgs195 22678114 PMC3514897

[49] Backlund MG Mann JR Holla VR Buchanan FG Tai HH Musiek ES . 15-hydroxyprostaglandin dehydrogenase is down-regulated in colorectal cancer. J Biol Chem. (2005) 280:3217–23. doi: 10.1074/jbc.m411221200 15542609 PMC1847633

[50] Yan M Myung SJ Fink SP Lawrence E Lutterbaugh J Yang P . 15-hydroxyprostaglandin dehydrogenase inactivation as a mechanism of resistance to celecoxib chemoprevention of colon tumors. Proc Natl Acad Sci USA. (2009) 106:9409–13. doi: 10.1073/pnas.0902367106 19470469 PMC2695050

[51] Solomon SD Mcmurray JJV Pfeffer MA Wittes J Fowler R Finn P . Cardiovascular risk associated with celecoxib in a clinical trial for colorectal adenoma prevention. N Engl J Med. (2005) 352:1071–80. doi: 10.1016/j.accreview.2005.05.024 15713944

[52] Gupta GP Nguyen DX Chiang AC Bos PD Kim JY Nadal C . Mediators of vascular remodelling co-opted for sequential steps in lung metastasis. Nature. (2007) 446:765. doi: 10.1038/nature05760 17429393

[53] Hill R Li Y Tran LM Dry S Calvopina JH Garcia A . Cell intrinsic role of COX-2 in pancreatic cancer development. Mol Cancer Ther. (2012) 11:2127–37. doi: 10.1158/1535-7163.mct-12-0342 22784710 PMC3469770

[54] Dragovich T Burris HIII Loehrer P Von Hoff DD Chow S Stratton S . Gemcitabine plus celecoxib in patients with advanced or metastatic pancreatic adenocarcinoma: Results of a phase II trial. Am J Clin Oncol. (2008) 31. doi: 10.1097/coc.0b013e31815878c9 18391600

[55] El-Rayes BF Zalupski MM Shields AF Ferris AM Vaishampayan U Heilbrun LK . A phase II study of celecoxib, gemcitabine, and cisplatin in advanced pancreatic cancer. Invest New Drugs. (2005) 23:583–90. doi: 10.1007/s10637-005-1028-z 16034525

[56] Tucker ON Dannenberg AJ Yang EK Zhang F Teng L Daly JM . Cyclooxygenase-2 expression is up-regulated in human pancreatic cancer. Cancer Res. (1999) 59:987–90. 10070951

[57] Kong G Kim EK Kim WS Lee KT Lee YW Lee JK . Role of cyclooxygenase-2 and inducible nitric oxide synthase in pancreatic cancer. J Gastroenterol Hepatol. (2002) 17:914–21. doi: 10.1046/j.1440-1746.2002.02829.x 12164968

[58] Yip-Schneider M Barnard D Billings S Cheng L Heilman D Lin A . Cyclooxygenase-2 expression in human pancreatic adenocarcinomas. Carcinogenesis. (2000) 21:139–46. doi: 10.1093/carcin/21.2.139 10657949

[59] Molina MA Sitja-Arnau M Lemoine G Frazier ML Sinicrope FA . Increased cyclooxygenase-2 expression in human pancreatic carcinomas and cell lines: Growth inhibition by nonsteroidal anti-inflammatory drugs. Cancer Res. (1999) 59:4356–62. 10485483

[60] Koshiba T Hosotani R Miyamoto Y Wada M Lee J Fujimoto K . Immunohistochemical analysis of cyclooxygenase-2 expression in pancreatic tumors. Int J Pancreatol. (1999) 26:69–76. doi: 10.1007/bf02781733 10597402

[61] Sato N Maehara N Goggins M . Gene expression profiling of tumor–stromal interactions between pancreatic cancer cells and stromal fibroblasts. Cancer Res. (2004) 64:6950–6. doi: 10.1158/0008-5472.can-04-0677 15466186

[62] Hwang D Byrne J Scollard D Levine E . Expression of cyclooxygenase-1 and cyclooxygenase-2 in human breast cancer. JNCI: J Natl Cancer Institute. (1998) 90:455–60. doi: 10.1093/jnci/90.6.455 9521170

[63] Crowell PL Schmidt CM Yip-Schneider MT Savage JJ Hertzler DA Cummings WO . Cyclooxygenase-2 expression in hamster and human pancreatic neoplasia. Neoplasia. (2006) 8:437–45. doi: 10.1593/neo.04700 16820089 PMC1601471

[64] Harris R Beebe-Donk J Alshafie GA . Cancer chemoprevention by cyclooxygenase 2 blockade: Results of case control studies. Sub-cell Biochem. (2007) 42:193–212. 10.1007/1-4020-5688-5_9 17612052

[65] Paget S . The distribution of secondary growths in cancer of the breast. 1889. Cancer Metastasis Rev. (1989) 8:98–101. doi: 10.1016/s0140-6736(00)49915-0 2673568

[66] Raskov H Orhan A Salanti A Gögenur I . Premetastatic niches, exosomes and circulating tumor cells: Early mechanisms of tumor dissemination and the relation to surgery. Int J Cancer. (2020) 146:3244–55. doi: 10.1002/ijc.32820 31808150

[67] Psaila B Zhang H Hiratsuka S Hoshino A Ghajar CM Kaplan RN . Pre-metastatic niches: Organ-specific homes for metastases. Nat Rev Cancer. (2017) 17:302–17. doi: 10.1038/nrc.2017.6 28303905

[68] Katoh H Hosono K Ito Y Suzuki T Ogawa Y Kubo H . COX-2 and prostaglandin EP3/EP4 signaling regulate the tumor stromal proangiogenic microenvironment via CXCL12-CXCR4 chemokine systems. Am J Pathol. (2010) 176:1469–83. doi: 10.2353/ajpath.2010.090607 20110411 PMC2832166

[69] Trinath J Hegde P Sharma M Maddur MS Rabin M Vallat J-M . Intravenous immunoglobulin expands regulatory T cells via induction of cyclooxygenase-2–dependent prostaglandin E2 in human dendritic cells. Blood. (2013) 122:1419–27. doi: 10.1182/blood-2012-11-468264 23847198

[70] Gialeli C Theocharis AD Karamanos NK . Roles of matrix metalloproteinases in cancer progression and their pharmacological targeting. FEBS J. (2011) 278:16–27. doi: 10.1111/j.1742-4658.2010.07919.x 21087457

[71] Bu X Zhao C Dai X . Involvement of COX-2/PGE(2) pathway in the upregulation of MMP-9 expression in pancreatic cancer. Gastroenterol Res Pract. (2011) 2011:214269. 21760774 10.1155/2011/214269 PMC3132487

[72] Cheng X-B Kohi S Koga A Hirata K Sato N . Hyaluronan stimulates pancreatic cancer cell motility. Oncotarget. (2015) 7:4829–40. doi: 10.1016/j.pan.2016.06.251 26684359 PMC4826246

[73] Chen S Chen X Li W Shan T Lin WR Ma J . Conversion of epithelial-to-mesenchymal transition to mesenchymal-to-epithelial transition is mediated by oxygen concentration in pancreatic cancer cells. Oncol Lett. (2018) 15:7144–52. doi: 10.3892/ol.2018.8219 29731878 PMC5921234

[74] Misra S Obeid LM Hannun YA Minamisawa S Berger FG Markwald RR . Hyaluronan constitutively regulates activation of COX-2-mediated cell survival activity in intestinal epithelial and colon carcinoma cells. J Biol Chem. (2008) 283:14335–44. doi: 10.1074/jbc.m703811200 18326857 PMC2386915

[75] Hosseini F Mahdian-Shakib A Jadidi-Niaragh F Enderami SE Mohammadi H Hemmatzadeh M . Anti‐inflammatory and anti‐tumor effects of α-l-guluronic acid (G2013) on cancer-related inflammation in a murine breast cancer model. Biomedicine Pharmacotherapy. (2018) 98:793–800. doi: 10.1016/j.biopha.2017.12.111 29571248

[76] Xie C Xu X Wang X Wei S Shao L Chen J . Cyclooxygenase-2 induces angiogenesis in pancreatic cancer mediated by prostaglandin E(2). Oncol Lett. (2018) 16:940–8. doi: 10.3892/ol.2018.8786 29963167 PMC6019925

[77] Ben-Batalla I Cubas-Cordova M Udonta F Wroblewski M Waizenegger JS Janning M . Cyclooxygenase-2 blockade can improve efficacy of VEGF-targeting drugs. Oncotarget. (2015) 6:6341–58. doi: 10.18632/oncotarget.3437 25849942 PMC4467441

[78] Holt D Ma X Kundu N Fulton A . Prostaglandin E(2) (PGE (2)) suppresses natural killer cell function primarily through the PGE(2) receptor EP4. Cancer Immunol Immunother. (2011) 60:1577–86. doi: 10.1007/s00262-011-1064-9 21681369 PMC3686482

[79] Dubey P Shrivastava R Tripathi C Jain N Tewari B Lone M . Cyclooxygenase-2 inhibition attenuates hypoxic cancer cells induced m2-polarization of macrophages. Cell Mol Biol. (2014) 60:10–5. 25210855

[80] Paik JH Ju JH Lee JY Boudreau MD Hwang DH . Two opposing effects of non-steroidal anti-inflammatory drugs on the expression of the inducible cyclooxygenase. Mediation through different signaling pathways. J Biol Chem. (2000) 275:28173–9. doi: 10.1074/jbc.m002329200 10866999

[81] Filipazzi P Valenti R Huber V Pilla L Canese P Iero M . Identification of a new subset of myeloid suppressor cells in peripheral blood of melanoma patients with modulation by a granulocyte-macrophage colony-stimulation factor–based antitumor vaccine. J Clin Oncol. (2007) 25:2546–53. doi: 10.1200/jco.2006.08.5829 17577033

[82] Hoechst B Ormandy LA Ballmaier M Lehner F Krüger C Manns MP . A new population of myeloid-derived suppressor cells in hepatocellular carcinoma patients induces CD4^+^CD25^+^Foxp3^+^ T cells. Gastroenterology. (2008) 135:234–43. doi: 10.1053/j.gastro.2008.03.020 18485901

[83] Rodriguez PC Quiceno DG Zabaleta J Ortiz B Zea AH Piazuelo MB . Arginase I production in the tumor microenvironment by mature myeloid cells inhibits T-cell receptor expression and antigen-specific T-cell responses. Cancer Res. (2004) 64:5839–49. doi: 10.1158/0008-5472.can-04-0465 15313928

[84] Serafini P Mgebroff S Noonan K Borrello I . Myeloid-derived suppressor cells promote cross-tolerance in B-cell lymphoma by expanding regulatory T cells. Cancer Res. (2008) 68:5439–49. doi: 10.1158/0008-5472.can-07-6621 18593947 PMC2887390

[85] Gantt S Gervassi A Jaspan H Horton H . The role of myeloid-derived suppressor cells in immune ontogeny. Front Immunol. (2014) 5:387. doi: 10.3389/fimmu.2014.00387 25165466 PMC4131407

[86] Monu NR Frey AB . Myeloid-derived suppressor cells and anti-tumor T cells: a complex relationship. Immunol Invest. (2012) 41:595–613. doi: 10.3109/08820139.2012.673191 23017137 PMC3701882

[87] Veltman JD Lambers MEH van Nimwegen M Hendriks RW Hoogsteden HC Aerts JGJV . COX-2 inhibition improves immunotherapy and is associated with decreased numbers of myeloid-derived suppressor cells in mesothelioma. Celecoxib influences MDSC function. BMC Cancer. (2010) 10:464. doi: 10.1186/1471-2407-10-464 20804550 PMC2939552

[88] Li YJ Kanaji N Wang XQ Sato T Nakanishi M Kim M . Prostaglandin E2 switches from a stimulator to an inhibitor of cell migration after epithelial-to-mesenchymal transition. Prostaglandins Other Lipid Mediat. (2015) 116–117:1–9. doi: 10.1016/j.prostaglandins.2014.10.003 25460827

[89] Sodergren MH Mangal N Wasan H Sadanandam A Balachandran VP Jiao LR . Immunological combination treatment holds the key to improving survival in pancreatic cancer. J Cancer Res Clin Oncol. (2020) 146:2897–911. doi: 10.1007/s00432-020-03332-5 32748119 PMC7519893

[90] Markosyan N Li J Sun YH Richman LP Lin JH Yan F . Tumor cell–intrinsic EPHA2 suppresses antitumor immunity by regulating PTGS2 (COX-2). J Clin Invest. (2019) 129:3594–609. doi: 10.1172/jci127755 31162144 PMC6715369

[91] Cook PJ Thomas R Kingsley PJ Shimizu F Montrose DC Marnett LJ . Cox-2-derived PGE2 induces Id1-dependent radiation resistance and self-renewal in experimental glioblastoma. Neuro Oncol. (2016) 18:1379–89. doi: 10.1093/neuonc/now049 27022132 PMC5035519

[92] Zhang N Yantiss RK Nam HS Chin Y Zhou XK Scherl EJ . ID1 is a functional marker for intestinal stem and progenitor cells required for normal response to injury. Stem Cell Rep. (2014) 3:716–24. doi: 10.1016/j.stemcr.2014.09.012 25418719 PMC4235234

[93] Zeng G Germinaro M Micsenyi A Monga NK Bell A Sood A . Aberrant Wnt/beta-catenin signaling in pancreatic adenocarcinoma. Neoplasia. (2006) 8:279–89. doi: 10.1593/neo.05607 16756720 PMC1600679

[94] van de Wetering M Sancho E Verweij C de Lau W Oving I Hurlstone A . The β-catenin/TCF-4 complex imposes a crypt progenitor phenotype on colorectal cancer cells. Cell. (2002) 111:241–50. doi: 10.1016/s0092-8674(02)01014-0 12408868

[95] Zhan T Rindtorff N Boutros M . Wnt signaling in cancer. Oncogene. (2017) 36:1461–73. doi: 10.1038/onc.2016.304 27617575 PMC5357762

[96] Vermeulen L De Sousa E Melo F van der Heijden M Cameron K de Jong JH Borovski T . Wnt activity defines colon cancer stem cells and is regulated by the microenvironment. Nat Cell Biol. (2010) 12:468–76. doi: 10.1038/ncb2048 20418870

[97] Wu M Guan J Li C Gunter S Nusrat L Ng S . Aberrantly activated Cox-2 and Wnt signaling interact to maintain cancer stem cells in glioblastoma. Oncotarget. (2017) 8:82217–30. doi: 10.18632/oncotarget.19283 29137258 PMC5669884

[98] Goessling W North TE Loewer S Lord AM Lee S Stoick-Cooper CL . Genetic interaction of PGE2 and Wnt signaling regulates developmental specification of stem cells and regeneration. Cell. (2009) 136:1136–47. doi: 10.1016/j.cell.2009.01.015 19303855 PMC2692708

[99] Nuñez F Bravo S Cruzat F Montecino M De Ferrari GV . Wnt/β-catenin signaling enhances cyclooxygenase-2 (COX2) transcriptional activity in gastric cancer cells. PloS One. (2011) 6:e18562. doi: 10.1371/journal.pone.0018562 21494638 PMC3071840

[100] Lu W Tinsley HN Keeton A Qu Z Piazza GA Li Y . Suppression of Wnt/beta-catenin signaling inhibits prostate cancer cell proliferation. Eur J Pharmacol. (2009) 602:8–14. doi: 10.1016/j.ejphar.2008.10.053 19026633 PMC2637082

[101] Zhang Z Wang J Dong X . Wnt2 contributes to the progression of gastric cancer by promoting cell migration and invasion. Oncol Lett. (2018) 16:2857–64. doi: 10.3892/ol.2018.9050 30127872 PMC6096226

[102] Fu L Zhang C Zhang LY Dong SS Lu LH Chen J . Wnt2 secreted by tumour fibroblasts promotes tumour progression in oesophageal cancer by activation of the Wnt/β-catenin signalling pathway. Gut. (2011) 60:1635–43. doi: 10.1136/gut.2011.241638 21672941

[103] Jiang H Li Q He C Li F Sheng H Shen X . Activation of the Wnt pathway through Wnt2 promotes metastasis in pancreatic cancer. Am J Cancer Res. (2014) 4:537–44. PMC4163618 25232495

[104] Prima V Kaliberova LN Kaliberov S Curiel DT Kusmartsev S . COX2/mPGES1/PGE2 pathway regulates PD-L1 expression in tumor-associated macrophages and myeloid-derived suppressor cells. Proc Natl Acad Sci USA. (2017) 114:1117–22. doi: 10.1073/pnas.1612920114 28096371 PMC5293015

[105] Raskov H Orhan A Gaggar S Gögenur I . Neutrophils and polymorphonuclear myeloid-derived suppressor cells: An emerging battleground in cancer therapy. Oncogenesis. (2022) 11. doi: 10.1038/S41389-022-00398-3 35504900 PMC9065109

[106] Liu B Qu L Yan S . Cyclooxygenase-2 promotes tumor growth and suppresses tumor immunity. Cancer Cell Int. (2015) 15:106. doi: 10.1186/s12935-015-0260-7 26549987 PMC4635545

[107] Gualde N Harizi H . Prostanoids and their receptors that modulate dendritic cell-mediated immunity. Immunol Cell Biol. (2004) 82:353–60. doi: 10.1111/j.0818-9641.2004.01251.x 15283844

[108] Göbel C Breitenbuecher F Kalkavan H Hähnel PS Kasper S Hoffarth S . Functional expression cloning identifies COX-2 as a suppressor of antigen-specific cancer immunity. Cell Death Dis. (2014) 5:e1568–e1568. doi: 10.1038/cddis.2014.531 25501829 PMC4649842

[109] Martinet L Jean C Dietrich G Fournié J-J Poupot R . PGE2 inhibits natural killer and γδ T cell cytotoxicity triggered by NKR and TCR through a cAMP-mediated PKA type I-dependent signaling. Biochem Pharmacol. (2010) 80:838–45. doi: 10.1016/j.bcp.2010.05.002 20470757

[110] Elder DJ Halton DE Hague A Paraskeva C . Induction of apoptotic cell death in human colorectal carcinoma cell lines by a cyclooxygenase-2 (COX-2)-selective nonsteroidal anti-inflammatory drug: independence from COX-2 protein expression. Clin Cancer Res. (1997) 3:1679–83. 9815550

[111] Groesch S Tegeder I Niederberger E Braeutigam L Geisslinger G . COX-2 independent induction of cell cycle arrest and apoptosis in colon cancer cells by the selective COX-2 inhibitor celecoxib. FASEB J. (2001) 15:2742–4. doi: 10.1096/fj.01-0299fje 11606477

[112] Zhang X Morham SG Langenbach R Young DA . Malignant transformation and antineoplastic actions of nonsteroidal antiinflammatory drugs (NSAIDs) on cyclooxygenase-null embryo fibroblasts. J Exp Med. (1999) 190:451–9. doi: 10.1084/jem.190.4.451 10449516 PMC2195603

[113] Lim JT Piazza GA Han EK Delohery TM Li H Finn TS . Sulindac derivatives inhibit growth and induce apoptosis in human prostate cancer cell lines. Biochem Pharmacol. (1999) 58:1097–107. doi: 10.1016/s0006-2952(99)00200-2 10484067

[114] Goluboff ET Prager D Rukstalis D Giantonio B Madorsky M Barken I . Safety and efficacy of exisulind for treatment of recurrent prostate cancer after radical prostatectomy. J Urol. (2001) 166:882. doi: 10.1016/S0022-5347(05)65856-9 11490238

[115] Thompson WJ Piazza GA Li H Liu L Fetter J Zhu B . Exisulind induction of apoptosis involves guanosine 3’,5’-cyclic monophosphate phosphodiesterase inhibition, protein kinase G activation, and attenuated beta-catenin. Cancer Res. (2000) 60:3338–42. 10910034

[116] Soh JW Mao Y Kim MG Pamukcu R Li H Piazza GA . Cyclic GMP mediates apoptosis induced by sulindac derivatives via activation of c-Jun NH2-terminal kinase 1. Clin Cancer Res. (2000) 6:4136–41. 11051267

[117] Yin M-J Yamamoto Y Gaynor RB . The anti-inflammatory agents aspirin and salicylate inhibit the activity of IκB kinase-β. Nature. (1998) 396:77. doi: 10.1038/23948 9817203

[118] Zhang L Yu J Park BH Kinzler KW Vogelstein B . Role of BAX in the apoptotic response to anticancer agents. Science. (2000) 290:989–92. doi: 10.1126/science.290.5493.989 11062132

[119] He T-C Chan T Vogelstein B Kinzler K . PPAR delta is an APC-regulated target of nonsteroidal anti-inflammatory drugs. Cell. (1999) 99:335–45. doi: 10.1016/s0092-8674(00)81664-5 10555149 PMC3779681

[120] Park BH Vogelstein B Kinzler KW . Genetic disruption of PPARδ decreases the tumorigenicity of human colon cancer cells. Proc Natl Acad Sci USA. (2001) 98:2598–603. doi: 10.1073/pnas.051630998 11226285 PMC30184

[121] Abdollahi A Schwager C Kleeff J Esposito I Domhan S Peschke P . Transcriptional network governing the angiogenic switch in human pancreatic cancer. Proc Natl Acad Sci USA. (2007) 104:12890–5. doi: 10.1073/pnas.0705505104 17652168 PMC1931565

[122] Williams CS Tsujii M Reese J Dey SK Dubois RN . Host cyclooxygenase-2 modulates carcinoma growth. J Clin Invest. (2000) 105:1589. doi: 10.1172/jci9621 10841517 PMC300858

[123] Williams CS Watson AJ Sheng H Helou R Shao J Dubois RN . Celecoxib prevents tumor growth *in vivo* without toxicity to normal gut: lack of correlation between *in vitro* and *in vivo* models. Cancer Res. (2000) 60:6045. 11085526

[124] Ben - Av P Crofford LJ Wilder RL Hla T . Induction of vascular endothelial growth factor expression in synovial fibroblasts by prostaglandin E and interleukin‐1: a potential mechanism for inflammatory angiogenesis. FEBS Lett. (1995) 372:83–7. doi: 10.1016/0014-5793(95)00956-a 7556649

[125] Bouvet M Ellis L Nishizaki M Fujiwara T Liu W Bucana C . Adenovirus-mediated wild-type p53 gene transfer down-regulates vascular endothelial growth factor expression and inhibits angiogenesis in human colon cancer. Cancer Res. (1998) 58:2288–92. 9622060

[126] Subbaramaiah K Altorki N Chung WJ Mestre JR Sampat A Dannenberg AJ . Inhibition of cyclooxygenase-2 gene expression by p53. J Biol Chem. (1999) 274:10911–5. doi: 10.1074/jbc.274.16.10911 10196169

[127] Hanahan D Weinberg R . Hallmarks of cancer: the next generation. Cell Press. (2011) 144:646–74. doi: 10.1016/j.cell.2011.02.013 21376230

[128] Peulen O Gonzalez A Peixoto P Turtoi A Mottet D Delvenne P . The anti-tumor effect of HDAC inhibition in a human pancreas cancer model is significantly improved by the simultaneous inhibition of cyclooxygenase 2. PloS One. (2013) 8:e75102–e75102. doi: 10.1371/journal.pone.0075102 24040391 PMC3770617

[129] Dai P Li J Ma X-P Huang J Meng J-J Gong P . Efficacy and safety of COX-2 inhibitors for advanced non-small-cell lung cancer with chemotherapy: a meta-analysis. Onco Targets Ther. (2018) 11:721–30. doi: 10.2147/ott.s148670 29440919 PMC5804138

[130] Erxleben A . Mitochondria-targeting anticancer metal complexes. Curr Med Chem. (2019) 26:694–728. doi: 10.2174/0929867325666180307112029 29521200

[131] Yasir Khan H Parveen S Yousuf I Tabassum S Arjmand F . Metal complexes of NSAIDs as potent anti-tumor chemotherapeutics: mechanistic insights into cytotoxic activity via multiple pathways primarily by inhibition of COX–1 and COX–2 enzymes. Coord Chem Rev. (2022) 453:214316. doi: 10.1016/j.ccr.2021.214316 38826717

[132] Martling A Hed Myrberg I Nilbert M Grönberg H Granath F Eklund M . Low-dose aspirin for PI3K-altered localized colorectal cancer. N Engl J Med. (2025) 393:1051–64. doi: 10.1056/nejmoa2504650 40961426

[133] Lévesque LE Brophy JM Zhang B . Time variations in the risk of myocardial infarction among elderly users of COX-2 inhibitors. CMAJ. (2006) 174:1563–9. doi: 10.1503/cmaj.051679 PMC1459899 16670396

[134] Lévesque LE Brophy JM Zhang B . The risk for myocardial infarction with cyclooxygenase-2 inhibitors: a population study of elderly adults. Ann Intern Med. (2005) 142:481–9. doi: 10.7326/0003-4819-142-7-200504050-00113 15809459

[135] FitzGerald GA . COX-2 and beyond: approaches to prostaglandin inhibition in human disease. Nat Rev Drug Discov. (2003) 2:879–90. doi: 10.1038/nrd1225 14668809

[136] Grosser T Ricciotti E FitzGerald GA . The cardiovascular pharmacology of nonsteroidal anti-inflammatory drugs. Trends Pharmacol Sci. (2017) 38:733–48. doi: 10.1016/j.tips.2017.05.008 28651847 PMC5676556

